# Characterization of *Staphylococcus aureus* from Distinct Geographic Locations in China: An Increasing Prevalence of *spa*-t030 and SCC*mec* Type III

**DOI:** 10.1371/journal.pone.0096255

**Published:** 2014-04-24

**Authors:** Yong Chen, Zhengxiang Liu, Libo Duo, Jie Xiong, Yanwen Gong, Jiyong Yang, Zhanke Wang, Xuqin Wu, Zhongyi Lu, Xiangzhao Meng, Jingya Zhao, Changjian Zhang, Fang Wang, Yulong Zhang, Mengqiang Zhang, Li Han

**Affiliations:** 1 Center for Hospital Infection Control, Chinese PLA Institute for Disease Control & Prevention, Academy of Military Medical Sciences, Beijing, China; 2 Department of Clinical Microbiology, Urumqi General Hospital of Lanzhou Military Command, PLA, Urumqi, China; 3 Department of Clinical Microbiology, The Second Affiliated Hospital of Harbin Medical University, Harbin, China; 4 Department of Clinical Laboratory, General Hospital of Chengdu Military Command, Chengdu, China; 5 Department of Clinical Laboratory, General Hospital of Ji'nan Military Command, Ji'nan, China; 6 Department of Clinical Microbiology, Chinese PLA General Hospital, Beijing, China; 7 Department of Clinical Laboratory, 94 Hospital of PLA, Nanchang, China; 8 Department of Clinical Laboratory, The First Affiliated Hospital of Soochow University, Suzhou, China; Rockefeller University, United States of America

## Abstract

*Staphylococcus aureus* belongs to one of the most common bacteria causing healthcare and community associated infections in China, but their molecular characterization has not been well studied. From May 2011 to June 2012, a total of 322 non-duplicate *S. aureus* isolates were consecutively collected from seven tertiary care hospitals in seven cities with distinct geographical locations in China, including 171 methicillin sensitive *S. aureus* (MSSA) and 151 MRSA isolates. All isolates were characterized by *spa* typing. The presence of virulence genes was tested by PCR. MRSA were further characterized by SCC*mec* typing. Seventy four and 16 *spa* types were identified among 168 MSSA and 150 MRSA, respectively. One *spa* type t030 accounted for 80.1% of all MRSA isolates, which was higher than previously reported, while *spa*-t037 accounted for only 4.0% of all MRSA isolates. The first six *spa* types (t309, t189, t034, t377, t078 and t091) accounted for about one third of all MSSA isolates. 121 of 151 MRSA isolates (80.1%) were identified as SCC*mec* type III. *pvl* gene was found in 32 MSSA (18.7%) and 5 MRSA (3.3%) isolates, with ST22-MSSA-t309 as the most commonly identified strain. Compared with non-epidemic MRSA clones, epidemic MRSA clones (corresponding to ST239) exhibited a lower susceptibility to rifampin, ciprofloxacin, gentamicin and trimethoprim-sulfamethoxazole, a higher prevalence of *sea* gene and a lower prevalence of *seb*, *sec*, *seg*, *sei* and *tst* genes. The increasing prevalence of multidrug resistant *spa*-t030 MRSA represents a major public health problem in China.

## Introduction

As one of the most important antibiotic-resistant pathogen in many parts of the world, the rates of methicillin resistant *Staphylococcus aureus* (MRSA) have been swiftly increasing worldwide over the past decades [1]. A review of the data from two main antimicrobial resistance surveillance programs (Mohnarin and CHINET) in China suggested that the proportion of MRSA among clinical *S. aureus* isolates increased dramatically from 20% in 1980 to 60% in 2008 [2]. Another Gram-Positive Cocci Resistance Surveillance program conducted in 12 teaching hospitals across China showed that the prevalence of MRSA dropped from 53.9% in 2005 to 48.1% in 2010, which might be due to the enhancement of infection control policy recently in China [3]. However, the high incidence of MRSA infection still represents a major problem in many hospital settings.

The evidence of many large molecular epidemiological studies showed that a limited number of predominant clones were responsible for the high prevalence of MRSA [4]–[6]. Only five *spa* types accounted for almost half (48.1%) of all MRSA isolates causing invasive infections in 26 European countries [6]. One multiply antibiotic resistance clone, defined by multilocus sequence typing (MLST) as ST239, is now widely disseminated in many Asian countries and China in particular [4], [7], [8], although it might be originated from European countries [9], [10]. ST239 is highly associated with the staphylococcal chromosomal cassette *mec* (SCC*mec*) type III genetic element and mainly corresponded to two *spa* types: t037 and t030 [4], [10]. Comparison of genome phylogeny with *spa* typing suggested that t037 represents the ancestral ST239 *spa* type [11], which is in accordance with the findings that t030 has replaced t037 as the most frequent MRSA type in China, although t037 still accounted for a high proportion of MRSA [12].

The characteristics of epidemic clones, their evolution and the reason for why some epidemic clones such as MRSA-ST239 are so common in hospitals in China are still unclear at present. A recent study found that a mobile genetic element–encoded gene, *sasX*, acted as a crucial factor promoting nasal colonization, virulence and a probable main driving force of the Asian MRSA epidemic [13]. In this study, we aimed to investigate the clinical, genetic characterizations of MRSA and methicillin sensitive *S. aureus* (MSSA) isolates representing different geographic origins in China during 2011 and 2012, and compare the prevalence of antibiotic resistance and virulence determinants including *pvl* and *sasX* genes among MRSA epidemic clones, non-epidemic clones and MSSA isolates.

## Materials and Methods

### Clinical isolates

From May 2011 to June 2012, a multicenter antibiotic-resistant bacteria surveillance program integrated by 8 clinical laboratories in China has been performed. A total of 322 non-duplicate *staphylococcus aureus* (*S. aureus*) isolates from different patients were consecutively collected from seven tertiary care hospitals in seven cities, which are located in distinct geographies in China [Northeast (Harbin), North (Beijing, Ji'nan), Northwest (Urumqi), Central (Nanchang), East (Suzhou), Southwest (Chengdu)] (Figure S1 of the supporting information). The clinical sources of isolates included the respiratory tract (n = 76), wound (n = 57), skin and soft tissue (n = 44), blood culture (n = 35), body fluid (n = 16), drainage (n = 11), urine (n = 7) and other sources (n = 76), such as pus, cerebral spinal fluid, catheter, et al. Eight isolates were from the outpatients or emergency departments, 30 isolates were obtained from intensive care units, 284 isolates were from the other inpatients departments. All the confirmed *S. aureus* isolates were sent to Chinese PLA Institute for Disease Control & Prevention in Beijing, who was responsible for organizing this surveillance program and conducting the molecular experiments.

### Antimicrobial susceptibility testing

Antimicrobial susceptibility was determined by the disk diffusion or minimal inhibitory concentration (MIC) methods according to the guidelines of Clinical and Laboratory Standards Institute (CLSI) [14]. The antimicrobial agents tested included oxacillin (OX), penicillin (P), erythromycin (E), clindamycin (CLI), rifampin (RIF), ciprofloxacin (CIP), gentamicin (GM), tetracycline (TET), trimethoprim-sulfamethoxazole (SXT), flusidic acid (FD), linezolid (LZD), nitrofurantoin (F), teicoplanin (TEC) and vancomycin (VA) (Oxoid ltd, Basingstoke, Hants, Chicago). Zone diameters ≤18 mm for fusidic acid was considered to be resistant as suggested[15], the susceptibility results of the other drugs were interpreted according to CLSI [14]. *S. aureus* strain ATCC 25923 or ATCC 29213 were used as quality control. Multidrug resistance was defined as resistance to ≥4 classes of antibiotics tested.

### Molecular experiments

#### DNA extraction

DNA was extracted by adding a mixture of 60 µl of 10 mg/ml lysozyme and 60 µl of 10 mg/ml lysostaphin for efficient lysis according to manufactures' technical manual (Wizard® Genomic DNA Purification Kit, Promega Corporation, Madison, WI, USA). The DNA was used as the template in all PCRs described below.

#### Detection of *mecA* and *mecC* genes

All the *S. aureus* isolates were screened for the presence of *mecA* gene as previously described [16]. A sample of *mecA*-negative isolates was screened for the presence of *mecC* gene using a specific PCR method [17]. The isolates carrying *mecA* or *mecC* gene were identified as MRSA.

#### *spa* typing and MLST

*spa* typing was performed as described previously [18]. Purified *spa* PCR products were sequenced with Applied Biosystems 3730XL DNA sequencer, *spa* types and repeats were assigned by using the *spa* database website (http://www.ridom.de/spaserver). The *spa* types were clustered into *spa* clonal complexes using the algorithm BURP using the Ridom StaphType version 2.2 software (http://www.ridom.de), with the calculated cost between the members of a group being less than or equal to 4 [19]. Multiple locus sequence typing (MLST) were performed in some representative isolates with special *spa* clones or virulence determinants, each allele sequence profile and the sequence type were determined according to the MLST database (http://saureus.mlst.net).

#### SCC*mec* typing

The *mec* complex of all the MRSA isolates were typed by multiplex PCR (M-PCR) with 20 primers as described by Milheirico et al [20]. Staphylococcal chromosomal cassette *mec* (SCC*mec*) types I to V were identified by comparing the M-PCR banding patterns of the isolates to those of the following reference strains: NCTC10442 (SCC*mec* type I), N315 (SCC*mec* type II), 85/2082 (SCC*mec* type III), JCSC4744 (SCC*mec* type IV), and CQ12109 (SCC*mec* type V). Single PCR assays with primers pairs of the M-PCR and a sequence based *ccrB* typing [21], [22] were performed for the nontypeable isolates. Amplification of the cassette chromosome recombinase gene, *ccrC*, was additionally performed as previously described [23], with a modification of annealing temperature to 52°C. Some of the PCR products were sequenced and further compared against the NCBI non-redundant protein database using BLASTP.

#### Identification of virulence genes

The presence of *pvl* gene encoding the Panton-Valentine leukocidin (PVL) toxin, *tst* gene encoding the toxic shock syndrome toxin 1 (TSST-1), virulence-associated gene *sasX* and 11 other virulence genes *sea*-*see*, *seg*-*sej*, *eta* and *etb* was tested by PCR for all isolates with the primers described previously [13], [24], [25]. Reaction mixtures contained 5 ng chromosomal DNA, oligonucleotide primers (0.2 µM), 100 µM each deoxynucleotide triphosphates, 10×PCR buffer (Mg^2+^ Plus), and 1.25 U Taq polymerase (Takara Bio Inc., Kyoto, Japan) in a final volume of 50 µl. PCR products were amplified with the following conditions: 94°C for 5 min, followed by 30 cycles of 94°C for 40 s, 52°C for 40 s, 72°C for 40 s, and a final elongation step of 72°C for 5 min. 4 µL of PCR product was run on a 1% agarose gel. The reaction was recorded as positive if a single clear band with the correct size was present.

### Collection of clinical data

The isolates information and corresponding medical records of the patients were reviewed by the local microbiological workers. The following variables were collected according to the standard protocol: isolation date, origin of clinical specimen, patient demographics (gender and age), patient location within the hospital; underlying diseases; epidemiological context (hospital acquisition or hospital onset when *S. aureus* isolated >48 h after admission, community onset when the isolation date was within 48 h after admission), patient's live status 14 days after initial isolate.

The study was approved by the institutional ethics committees of the Academy of Military Medical Sciences of the Chinese People's Liberation Army, Beijing, China. As all data were anonymously collected and interpreted, the need for written informed consent from the participants was waived by the institutional ethics committees.

### Statistical analysis

Statistical analysis was carried out using SPSS 16.0 for Windows (SPSS inc., Chicago, IL, USA). Chi-square test and nonparametric test were used for comparing proportions and the median age, respectively, for the summary statistics. Fisher's exact test was used to compare the prevalence of antibiotic resistance and virulence factors as appropriate. The index of diversity of *spa* typing and the 95% confidence intervals (CIs) were calculated as described previously [26]. A *p* value of <0.05 was considered statistically significant.

## Results

### Isolates and patients

Among the 322 non-duplicate *S. aureus* collected from seven hospitals between May 2011 to June 2012, 171 (53.1%) and 151 (46.9%) isolates were identified as MSSA and MRSA, respectively (Table 1). All the MRSA isolates carried *mecA* gene and no *mecC*-positive isolates were found. The proportion of MRSA among *S. aureus* obtained from the ICU wards was significantly higher than that from other wards (86.7% VS 42.8%, *p*<0.001). Patients with MRSA colonization or infection were older with a median age of 50 y compared to MSSA patients with a median age of 45 y (*p* = 0.005). One hundred of 127 MRSA (78.7%) and 50 of 143 MSSA (35.0%) were reported as hospital onset isolates, respectively. The difference of hospital acquisition proportions between MRSA and MSSA isolates was significant (*p*<0.001). The gender distribution and all-cause mortality of patients 14 d after isolation of *S. aureus* did not differ between MSSA and MRSA (Table 2).

**Table 1:** Distribution of MSSA and MRSA isolates and their predominant *spa* types in cities throughout China.

City	No. of isolates	MSSA			MRSA
		No	Predominant *spa* types (no. of isolates)	No	Predominant *spa* types(no. of isolates)
Beijing	43	21	t034(3),t437(3)	22	t030(19)
Chengdu	55	35	t189(5),t377(5),t034(4)	20	t030(13)
Harbin	59	25	t078(7)	34	t030(32)
Ji'nan	50	32	t796(3)	18	t030(12)
Nanchang	27	12	t189(2)	15	t030(10)
Suzhou	12	3*	-	9	t030(5)
Urumqi	76	43	t309(14)	33	t030(30)
Total	322	171	t309(14),t189(11), t034(10)	151	t030(121)

*The number of predominant *spa* types was not calculated due to limited number of isolates.

**Table 2:** Summary statistics of patients and *spa* typing for MSSA/MRSA isolated in seven hospitals.

Statistics	*n* a	MSSA	MRSA	Total/Overall	?^2^	*p*-value^b^
Male gender (%)	321	112(65.9)	103(68.2)	215(67.0)	0.19	0.658
Median age (IQR)	320	45(26–59)	50(37–65)	47(32–64)	-	0.005
ICU wards (%)	322	4(2.3)	26(17.2)	30(9.3)	20.01	<0.001
Hospital acquisition (%)	270	50(35.0)	100(78.7)	150(55.6)	52.20	<0.001
No. *spa* types	318	74	16	81	-	-
No. not typeable	4	3	1	4	-	-
Index of diversity (95% CI)	318	0.975(0.968–0.983)	0.347(0.246–0.448)	0.844(0.804–0.884)	-	<0.05^c^
PVL(%)	322	32(18.7)	5(3.3)	37(11.5)	18.71	<0.001
All-cause mortality of patients 14 d after isolation of *S. aureus*	250	4(3.1)	8(6.7)	12(4.8)	1.80	0.180

a Number of isolates for which data were available.

b *p*-value for the comparison of MSSA versus MRSA.

c Deduced from non-overlapping 95% confidence intervals.

IQR, interquartile range.

### *spa* typing

A total of 81 *spa* types were assigned to 318 *S. aureus*, leaving 4 isolates not typeable. 168 MSSA contained 74 *spa* types, while 150 MRSA had 16 *spa* types. There were 9 *spa* types (t002, t030, t078, t127, t437, t701, t1376, t4549 and t5554) found in both MSSA and MRSA. The index of diversity (DI) for MSSA was 0.975 (95% CI: 0.968–0.983), which was significantly higher than for MRSA (DI = 0.347, 95% CI: 0.246–0.448) (Table 2). 121 and 6 MRSA isolates were identified as *spa* type t030 and t037, respectively, which accounted for 80.1% and 4.0% of all MRSA isolates, whereas the first six *spa* types (t309, t189, t034, t377, t078 and t091) accounted for 33.3% of all MSSA isolates. Four novel *spa* types (t12440, t12441, t12442, t12444) were identified firstly in MSSA in our study.

*spa*-t030 was uniformly predominant in MRSA isolates from all the seven hospitals, with the proportion ranging from 56% in a hospital in Suzhou to 94% in a hospital in Harbin (Figure S1). However, the predominant *spa* types in MSSA isolates differed largely from different hospitals. The most common MSSA isolates, *spa*-t309, were all from the hospital in Urumqi, while seven *spa*-t078 isolates were all from the hospital in Harbin, and *spa*-t189 were mainly indentified in isolates from the hospitals in Chengdu and Nanchang (Table 1). As shown in Table 3, *spa*-t030 was the most frequent *spa* type in *S. aureus* isolates of different sources, except for skin or soft tissue and urine. Among the *S. aureus* isolates from skin or soft tissue, t309 (n = 7), t437 (n = 5) and t796 (n = 3) were three most frequent *spa* types.

**Table 3:** The prevalence of MRSA, PVL and predominant *spa* types among different isolate sources.

Isolate sources	Total Number	MRSA no.(%)	PVL no.(%)	Predominant *spa* types (%)
Blood culture	35	17(48.6)	1(2.9)	t030(40.0), t078(8.6)
Body fluid	16	11(68.8)	2(12.5)	t030(68.8)
Drainage	11	8(72.7)	0(0.0)	t030(63.6)
Skin or soft tissue	44	5(11.4)	14(31.8)	t309(15.9), t437(11.4), t796(6.8)
Sputum	76	53(69.7)	1(1.3)	t030(61.8), t002(3.9), t034(3.9)
Urine	7	1(14.3)	1(14.3)	UN
Wound	57	31(54.4)	5(8.8)	t030(49.1), t189(7.0)
Others	76	25(32.9)	13(17.1)	t030(18.4), t437(7.9), t034(5.3), t002(3.9), t037(3.9)
Total	322	151(46.9)	37(11.5)	t030(37.9), t309(4.3), t437(4.0), t189(3.4), t034(3.1), t002(2.8)

UN: The numbers of different *spa* types were equally distributed.

**Figure 1:**
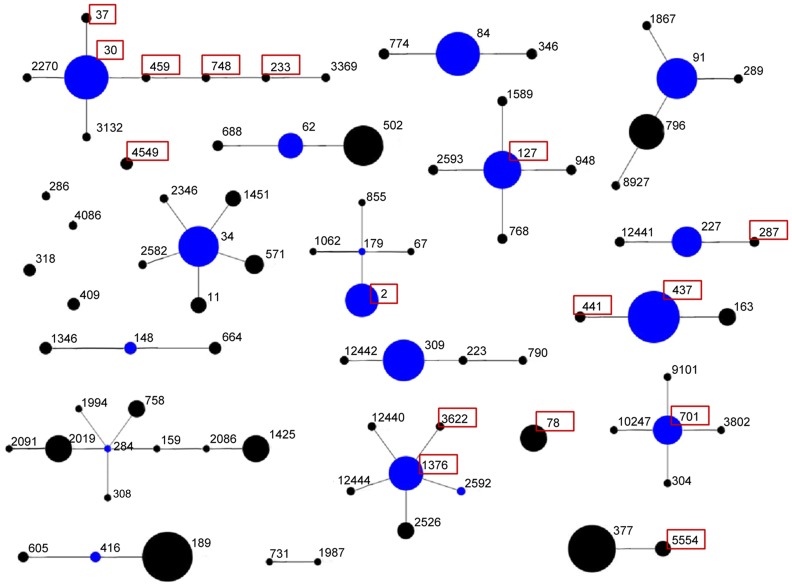
Population snapshot of 318 *Staphylococcus aureus* isolates described in the current study, using the results of *spa* typing. Clusters of linked isolates correspond to clonal complexes by means of the BURP algorithm. Primary founders (in blue) are positioned centrally in the cluster. The highlighted numbers indicate *spa* types identified in the MRSA populations.

BURP analysis of 318 typeable *S. aureus* strains showed that 301 strains were clustered into 17 *spa*-CCs. The four most common CCs were CC030, CC034, CC309 and CC437, which accounted for 42.1%, 6.9%, 5.3% and 5.3% of all the typeable strains, respectively. MSSA were found in all *spa*-CCs, while MRSA were found in only eight *spa*-CCs (Figure 1).

### SCC*mec* typing

Among the 151 MRSA isolates, four SCC*mec* types including II (3, 2.0%), III (121, 80,1%), IV (10, 6.6%), V (8, 5.3%) were identified, leaving 9 isolates (6.0%) classified as nontypeable (NT), although repeated singular PCR assay and *ccrB* typing were conducted. Three *spa*-t002 isolates from Suzhou were identified as SCC*mec* type II, which exhibited multidrug resistance patterns and carried multiple virulence genes. *spa*-t030 accounted for 91.7% of all the SCC*mec* type III isolates, which also exhibited multidrug resistance patterns and widely distributed in the seven hospitals. All the six *spa*-t037 MRSA were identified as SCC*mec* type III. Six of the seven *spa*-t437 MRSA were identified as SCC*mec* type IV (Table 4). The *ccrC* gene, which always lies in the left extremity of SCC*mec* elements, was identified in 120 of the 121 type III strains and all the type V strains. The *spa* type of the strain without *ccrC* was t078.

**Table 4:** The first five *spa* types, antibiotic resistance profile, virulence gene profile and distribution by city among MRSA and MSSA isolates.

MRSA or MSSA	*spa* type	No. of isolates (%)	Predominant Resistance phenotype (no. of isolates)^a^	Predominant virulence gene profile(no. of isolates)	Distribution by city (no. of isolates)^b^
MRSA	t030	121(80.1)	P,E,CLI,RIF,CIP,GM,TET (49)	*sea* (109)	BJ(19),CD(13),HB(32), JN(12),NC(10),SZ(5), Ur(30)
	t437	7(4.6)	P,E,CLI (6)	*pvl* (4)	JN(4),CD(2),NC(1)
	t037	6(4.0)	P,E,CLI,CIP,GM,TET (6)	*sea* (6)	NC(2),BJ(1),CD(1),JN(1),Ur(1)
	t002	3(2.0)	P,E,CLI,CIP,GM,TET (3)	*seb*,*sec*,*seg*,*sei*, *tst* (3)	SZ(3)
	t4549	2(1.3)	P(2)	-	CD(2)
MSSA	t309	14(8.2)	P,E, SXT(8)	*sea*,*pvl* (8)	Ur(14)
	t189	11(6.4)	P(10)	*seb*,*pvl* (2)	CD(5),JN(2),NC(2),BJ(1),Ur(1)
	t034	10(5.8)	P,E(8)	*sea* (4)	CD(4),BJ(3),JN(1),NC(1),Ur(1)
	t377	8(4.7)	P,E,CIP(5)	-	CD(5),BJ(1),JN(1),Ur(1)
	t078	7(4.1)	P,E,CIP,GM (4)	*sea*,*seg*,*sei* (6)	HB(7)

a P, penicillin; E, erythromycin; CLI, clindamycin; RIF, rifampin; CIP, ciprofloxacin; GM, gentamicin; TET, tetracycline; SXT, trimethoprim-sulfamethoxazole.

b BJ, Beijing; CD, Chengdu; HB, Harbin; JN, Ji'nan; NC, Nanchang; SZ, Suzhou; Ur, Urumqi.

NT: not typeable.

### Distribution of *pvl*, *sasX* and other virulence genes

Thirty-seven isolates (11.5% of all) were found positive for the presence of *pvl* gene, including 32 MSSA and 5 MRSA isolates. Of the 93 and 50 MSSA isolates that were reported as community and hospital onset isolates, 23(24.7%) and 6(12.0%) were *pvl*-positive, respectively. However, the difference was not significant (χ^2^ = 3.26, *p* = 0.071). Fourteen *pvl*-positive isolates were cultured from skin or soft tissue. The first four *spa* types of *pvl*-positive isolates were t309 (n = 9, 24.3%), t437 (n = 5, 13.5%), t011(n = 3, 8.1%) and t189 (n = 3, 8.1%). Three *pvl*-positive MRSA (*spa*-t437) were classified as SCC*mec* type IV, the other two (*spa*-t437 and *spa*-t287) were SCC*mec* type V.

Five MRSA isolated from four different sample sources (drainage, pus, sputum, body fluid) in four hospitals were found positive for the presence of *sasX* gene. Of them, three isolates were *spa*-t037 exhibiting resistance to all nine kinds of antibiotics tested and the other two were *spa*-t030 exhibiting also multidrug resistance pattern. All the five *sasX*-positive isolates belonged to SCC*mec* type III and carried *sea* virulence gene. The *sea* gene was identified with the proportion of 58.5% and 82.1% in MSSA and MRSA, respectively. The prevalence of *seb*-*sed*, *seh*, *eta*, *etb*, and *tst* genes in MSSA and MRSA were all less than 10%. The *eta* and *etb* genes were only found in MSSA isolates. No *see* gene was found in both MSSA and MRSA.

### MLST typing

To show *S. aureus* clones more clearly, seventy strains, including 12 *spa*-t030 strains, 4 *spa*-t037 strains, 17 other representative MRSA clones and all the 37 *pvl*-positive strains were chosen for MLST typing. All of the 16 *spa*-t030 and *spa*-t037 isolates tested (including five *sasX*-positive isolates), as well as three other *spa*-CC030 MRSA clones, *spa*-t233, *spa*-t459 and *spa*-t748 were identified as ST239, which represents epidemic MRSA clones in our study. Six *spa*-t437 and one *spa*-t441 MRSA clones were identified as ST59 (Table 5). Eleven ST types were indentified among the 37 *pvl*-positve *S. aureus* isolates. The first three ST types were ST22 (n = 10, 27.0%), ST398 (n = 8, 21.6%) and ST59 (n = 6, 16.2%). The detailed information of the 37 *pvl*-positve *S. aureus* isolates were shown in the supplemental information (Table S1).

**Table 5:** The distribution of MLST types, SCC*mec* types and virulence genes in different MRSA clones according to *spa* clone complex.

*spa*-CC^a^	*spa* type	No. (%) of isolates	MLST (no. of isolates tested)	MLST allele profile^b^	SCC*mec* type (no. of isolates)	Carriage of virulence genes (no. of isolates)
CC030	t030	121(80.1)	ST239(12)	2-3-1-1-4-4-3	?(111), IV(1), V(3), NT (6)	*sea*(109),*seb*(1),*sec*(2),*sed*(1),*seg*(8), *seh*(2), *sei*(2),*sej*(5), *tst*(1), *sasX*(2)
	t037	6(4.0)	ST239(4)	2-3-1-1-4-4-3	?(6)	*sea*(6), *sasX*(3)
	t233	1(0.7)	ST239(1)	2-3-1-1-4-4-3	?(1)	*sea*(1), *sec*(1)
	t459	1(0.7)	ST239(1)	2-3-1-1-4-4-3	?(1)	*sea*(1)
	t748	1(0.7)	ST239(1)	2-3-1-1-4-4-3	IV(1)	*sea*(1)
CC437	t437	7(4.6)	ST59(6)	19-23-15-2-19-20-15	IV(6), V(1)	*pvl*(4), *sea*(1), *seb*(4),*sec*(1), *sed*(1), *sej*(1)
	t441	1(0.7)	ST59(1)	19-23-15-2-19-20-15	IV(1)	None
CC002	t002	3(2.0)	ST764(2)	1-136-1-4-12-1-10	?(3)	*sea*(1), *seb*(3),*sec*(3), *seg*(3), *sei*(3),*tst*(3)
Singleton	t4549	2(1.3)	ST630(2)	12-3-1-1-4-4-3	V(2)	*sea*(1)
CC127	t127	1(0.7)	ST1(1)	1-1-1-1-1-1-1	IV(1)	*sea*(1), *seh*(1)
CC227	t287	1(0.7)	ST25(1)	4-1-4-1-5-5-4	V(1)	*pvl*(1), *seb*(1),*seg*(1), *sei*(1)
CC701	t701	1(0.7)	ST6(1)	12-4-1-4-12-1-3	NT (1)	None
CC1376	t1376	1(0.7)	ST1298(1)	22-1-14-109-12-4-31	NT (1)	*sea*(1)
	t3622	1(0.7)	ST88(1)	22-1-14-23-12-4-31	IV(1)	*sea*(1)
Singleton	t078	1(0.7)	NT(1)	1-1-4-1-new-new-1	?(1)	*seg*(1)
Unkown	t5554	1(0.7)	ST630 SLV	12-334-1-1-4-4-3	V(1)	None
	NT	1(0.7)	NT(1)	1-4-new-169-new-57-11	NT (1)	None

a CC: clone complex.

b Allelic profile in order of *arcC*-*aroE*-*glpF*-*gmk*-*pta*-*tpi*-*yqiL*.

SLV: single locus variant.

NT: not typeable, the reasons for the nontypeable of some isolates were mainly due to that a low quality of sequence chromatogram was present for *spa* typing, or at least one new allele was identified in the seven housekeeping genes for MLST typing, or no corresponding band was found in the multiplex PCR for SCC*mec* typing. The typing experiments were repeated at least twice for the nontypeable isolates.

### Comparisons of the prevalence of antibiotic resistance and presence of virulence genes

**Figure 2:**
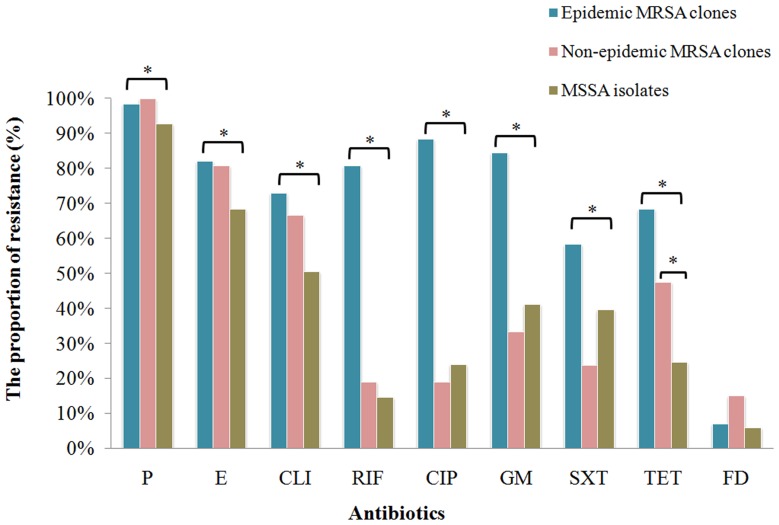
The comparisons of antibiotics resistance of epidemic MRSA clones (corresponding to ST239) and non-epidemic MRSA clones with MSSA isolates. Chi-square test and Fisher's exact test were used for comparison. P, penicillin; E, erythromycin; CLI, clindamycin; RIF, rifampin; CIP, ciprofloxacin; GM, gentamicin; TET, tetracycline; SXT, trimethoprim-sulfamethoxazole, FD, flusidic acid. * P<0.05.

The results of susceptibility testing showed that all the isolates tested were susceptible to vancomycin, teicoplanin and linezolid without exception and only 2 isolates (MRSA-t030 and MSSA-t2019) were resistant to nitrofurantoin. The resistance rates of other antibiotics ranged from 6.9% (flusidic acid) to 95.6% (penicillin). We divided MRSA isolates into epidemic clones group (all of the isolates corresponding to ST239 in our study) and non-epidemic clones group according to the results of *spa* typing and MLST. Compared with non-epidemic MRSA clones, epidemic MRSA clones exhibited a lower susceptibility to rifampin, ciprofloxacin, gentamicin and trimethoprim-sulfamethoxazole. Compared with MSSA, epidemic MRSA clones exhibited a lower susceptibility to eight kinds of antibiotics commonly used, while non-epidemic MRSA clones exhibited a lower susceptibility only to tetracycline (Figure 2, Table S2).

**Figure 3:**
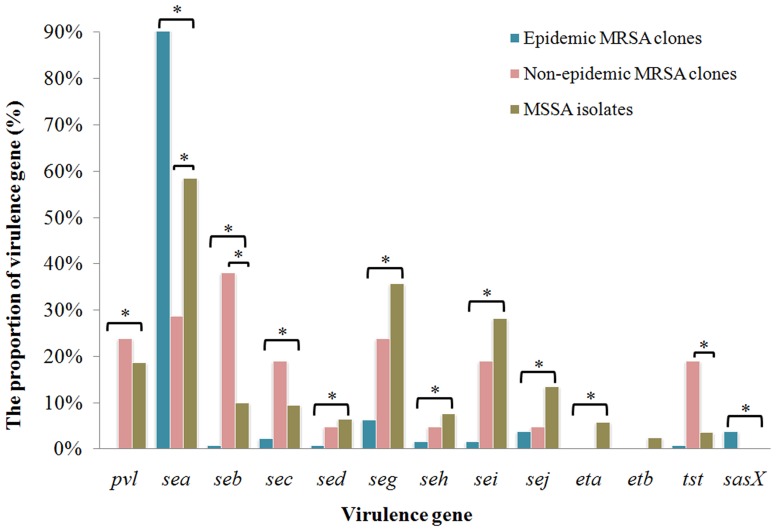
The comparisons of virulence genes distribution of epidemic MRSA clones (corresponding to ST239) and non-epidemic MRSA clones with MSSA isolates. Chi-square test and Fisher's exact test were used for comparison. * P<0.05.

Epidemic MRSA clones didn't carry *pvl* genes. Compared with both non-epidemic MRSA clones and MSSA, epidemic MRSA clones exhibited a higher prevalence of *sea* gene and a lower prevalence of *seb*, *sec*, *seg* and *sei* genes. Epidemic MRSA clones also had a lower prevalence of *sed*, *seh* and *sej* genes than MSSA. The non-epidemic MRSA clones exhibited a lower prevalence of *sea* as well as a higher prevalence of *seb* and *tst* genes than MSSA (Figure 3, Table S2).

## Discussion

*S. aureus* represents a substantial disease burden mainly related to health care in China. To the present, however, the molecular characterizations of clinical *S. aureus* isolates from China remain unclear, especially for MSSA isolates. *spa* typing, which has been showed to possess a higher discriminatory power than MLST [19], is one of the most widely molecular typing techniques for *S. aureus* in terms of easy, fast and comparable between different laboratories. In this study, we mainly used *spa* typing and MLST to illustrate the molecular characterizations of clinical *S. aureus* isolates in China.

In comparison to other studies [5], [6], [12], [27], our typing results indicated that different predominant clones circulated in certain districts and may change over time. In our study, *spa*-t030 accounted for 80.1% of all MRSA isolates and predominated in all the seven hospitals, which was generally in accordance with previous report, but with a much higher proportion [4], [28], [29]. One large study conducted in China found that ST5-MRSA-II-t002 was a major MRSA clone other than ST239 clones and accounted for nearly 16% of MRSA in China, *spa*-t002 predominated in Dalian and Shenyang in the northeast of China, *spa*-t037 predominated in Shanghai in the east of China [4]. However, in our study, only three and six MRSA were identified as t002 and t037, respectively. Although Shanghai was not contained in our study, it has been shown recently that Shanghai, Chongqing and Guangzhou were three major sources of MRSA-t002 and MRSA-t037 clones in China, while in other parts of China, including Shenyang, t030 was the only predominant *spa* type in MRSA [28], [30]. Our study strongly suggested that the prevalence rates of ST239-MRSA-t030 in Beijing, Harbin and Urumqi, which are all in the north of China, were especially high.

Another major MRSA clone *spa*-t037 accounted for only 4.0% of all MRSA isolates in our study. Therefore, it seemed that *spa*-t037 was continuously replaced by *spa*-t030 in China. A recent microarray-based comparative genomic study found that three gene clusters (vSa4, phage ?Sa1, and ?Sa3) were unique to *spa*-t030 and may contribute to its rapid replacement of *spa*-t037 [31]. But the exact determinants of this evolution process were still unclear, as whole genome analysis did not give any answer to questions concerning epidemic of MRSA clones so far. Although the *sasX* gene plays a key role in MRSA colonization and pathogenesis [13], it could not be the main reason for MRSA epidemic in clinical settings in China, as its prevalence was relatively low in both *spa*-t030 and *spa*-t037 isolates.

None of the four *spa* types (t032, t008, t041, t003) which accounted for 41.6% of invasive MRSA infection in Europe [6] were identified in our study. On the other hand, t008 and t002, which were the most common *spa* types among blood and nasal culture isolates of MRSA in USA [27]_,_ were rarely found in our study. Interestingly, two *spa*-t002 MRSA strains in our study corresponded to ST764, but not frequent ST5 in other studies [32]. *spa*-t437 strains, which were closely associated with community acquired skin and soft tissue infection [33], possessed a high prevalence in both MRSA and MSSA isolates, and all corresponded to ST59 in our study. This clone has been mainly reported in many Asian countries [34].

Among the first six *spa* types of MSSA isolates, t189 was also identified as predominant in Malaysian and China [35], [36], t034, which always corresponds to ST398, was characterized as livestock-associated isolates and prevalent among isolates from skin or soft tissue infection in many countries including China [37]–[39], t078 was recently found to be associated with community acquired skin and soft tissue infection [40], t091 has recently been showed to be a major genotype of MSSA isolated from bacteriaemia in China [41], t309 and t377 were seldom noted for Chinese isolates previously. In general, MSSA possessed a high genetic diversity, which suggested that the epidemic MRSA isolates were more likely to be imported or clone transmitted instead of arisen from successful MSSA clones and is in accordance with previous studies [6], [42].

The results of SCC*mec* typing showed that *spa*-t030 and t037 clones were typically associated with SCC*mec* type III, which constituted most of the noscomial MRSA and was responsible for the high prevalence of multidrug resistance in clinical MRSA isolates from China [7]. Six of the ten SCC*mec* type IV isolates (60.0%) were typed as *spa*-t437, which was the first predominant clone of community-associated MRSA isolates in Asian countries and showed high resistance rates to erythromycin and clindamycin [34].

PVL has been associated with community-associated or onset MRSA and tended to cause skin infections and severe necrotizing pneumonia [43].The prevalence of PVL among *S. aureus* isolates varied a lot with different genetic or epidemiological background or from different countries/regions. A multicenter study from Europe showed that 51% of community onset MRSA isolates were PVL-positive [44]. It has been reported that 43.2% of MRSA and 9.6% of MSSA isolates from blood culture in China were PVL-positive [35], However, another study showed that there was a high prevalence of PVL among MSSA isolates (41.5%) causing skin and soft tissue infection in Beijing, China [39]. In our study, the prevalence of PVL was significant higher among MSSA isolates than MRSA isolates (19.3% VS 3.4%, *p*<0.001), though both at a relatively low level. In addition, we found that *pvl* gene was more commonly identified among isolates from skin and soft tissue than from other sources, such as blood culture, sputum, urine, etc. It was not surprising to see that PVL was not limited to specific genetic backgrounds or clones due to the high diversity of MSSA isolates. However, we identified an unusually high prevalence of PVL-producing ST22-MSSA-t309 strains in a hospital form Urumqi in the Northwest of China for the first time (Table S1).

As a major cause of MRSA infections previously or currently in many Asian and European hospitals, including Singapore, Austria, Eastern Russia and Turkey [9], [45]–[47], ST239 has diversified into a clonal group including many *spa* types, such as t003, t030, t037, t221, t459, etc. [41], [45], [48]. Our classification of epidemic MRSA clones and non-epidemic MRSA clones corresponds to ST239 and non-ST239 group and is justified for the comparisons of the prevalence of antimicrobial resistance and virulence factors. Our analysis showed that the epidemic MRSA clones exhibited a higher resistance rate than non-epidemic MRSA clones over many commonly prescribed antibiotics, including rifampin and trimethoprim-sulfamethoxazole, which might be one important reason for the increasing prevalence of *spa*-t030 among MRSA isolates in China. However, nosocomial MRSA currently prevalent in Europe (ST22, ST225) are mostly resistant to β-lactams, fluoroquinolones, erythromycin, and clindamycin only, the same is applicable to MRSA ST5, which is prevalent in the USA [49], [50]. It suggests that epidemicity of hospital associated MRSA is not simply based on multiple resistances.

We also found that epidemic MRSA clones differed from non-epidemic MRSA clones in the distribution of many virulent determinants, including a higher prevalence of *sea* gene and a lower prevalence of *seb*, *sec*, *seg* and *sei* genes. When epidemic MRSA clones were excluded from the analysis, the difference in the prevalence of many virulent genes between MRSA and MSSA isolates became non-significant, which implies that non-epidemic MRSA clones were highly similar to MSSA in terms of virulence profiles and might have recently evolved from MSSA through horizontal transfer of some mobile genetic elements. However, it remains unclear why the epidemic MRSA clones, especially *spa*-t030, are so widely distributed in China and some other countries. One good explanation as described previously was that the multiplicity of cultural and social behaviors and habits, such as frequency and destination of travel, infection control measures and antibiotic prescription and consumption habits, may have shaped population structure of MRSA in these countries [44]. The high frequency of patient transfer between hospitals from different cities and abuse of antibiotics in China could contribute to the widely dissemination of *spa*-t030, but international medical travel between China and other countries is still not so common, which might help to prevent *spa*-t030 from spreading too fast. However, this situation may change in the near future.

In conclusion, our study provided a new genetic snapshot of *S. aureus* isolates from hospitals in distinct geographies in China, identified an increasing prevalence of *spa*-t030 strains, as well as illustrated many information about the clinical, resistance, and molecular characterization of clinical *S. aureus* isolates in China.

## Supporting Information

Figure S1 **Locations of seven participating hospitals in China and the proportions of** ***spa*** **-t030 and other** ***spa*** **types among MRSA isolates in this study.**
(TIF)

Table S1 **The corresponding spa types, number of MRSA, virulence genes distribution and city distribution of 37 pvl-positive isolates according to different ST types.**
(DOCX)

Table S2 Comparisons of the proportion of antibiotic resistance and presence of virulence factors among epidemic MRSA clones, non-epidemic MRSA clones and MSSA isolates^*^.
(DOCX)
