# Correction: Characterization of *Staphylococcus aureus* from Distinct Geographic Locations in China: An Increasing Prevalence of *spa*-t030 and SCC*mec* Type III

**DOI:** 10.1371/journal.pone.0112002

**Published:** 2014-10-22

**Authors:** The PLOS ONE Staff

In Table 5, there are multiple errors in the “SCC*mec* type (no. of isolates)” column. Please see the corrected Table 5 here.

**Table 5:** The distribution of MLST types, SCC*mec* types and virulence genes in different MRSA clones according to *spa* clone complex.

*spa*-CC a	*spa* type	No. (%) of isolates	MLST (no. of isolates tested)	MLST allele profile b	SCC*mec* type (no. of isolates)	Carriage of virulence genes (no. of isolates)
CC030	t030	121(80.1)	ST239(12)	2-3-1-1-4-4-3	III (111), IV(1), V(3), NT (6)	*sea*(109),*seb*(1),*sec*(2),*sed*(1),*seg*(8), *seh*(2), *sei*(2),*sej*(5), *tst*(1), *sasX*(2)
	t037	6(4.0)	ST239(4)	2-3-1-1-4-4-3	III (6)	*sea*(6), *sasX*(3)
	t233	1(0.7)	ST239(1)	2-3-1-1-4-4-3	III (1)	*sea*(1), *sec*(1)
	t459	1(0.7)	ST239(1)	2-3-1-1-4-4-3	III (1)	*sea*(1)
	t748	1(0.7)	ST239(1)	2-3-1-1-4-4-3	IV(1)	*sea*(1)
CC437	t437	7(4.6)	ST59(6)	19-23-15-2-19-20-15	IV(6), V(1)	*pvl*(4), *sea*(1), *seb*(4),*sec*(1), *sed*(1), *sej*(1)
	t441	1(0.7)	ST59(1)	19-23-15-2-19-20-15	IV(1)	None
CC002	t002	3(2.0)	ST764(2)	1-136-1-4-12-1-10	II (3)	*sea*(1), *seb*(3),*sec*(3), *seg*(3), *sei*(3),*tst*(3)
Singleton	t4549	2(1.3)	ST630(2)	12-3-1-1-4-4-3	V(2)	*sea*(1)
CC127	t127	1(0.7)	ST1(1)	1-1-1-1-1-1-1	IV(1)	*sea*(1), *seh*(1)
CC227	t287	1(0.7)	ST25(1)	4-1-4-1-5-5-4	V(1)	*pvl*(1), *seb*(1),*seg*(1), *sei*(1)
CC701	t701	1(0.7)	ST6(1)	12-4-1-4-12-1-3	NT (1)	None
CC1376	t1376	1(0.7)	ST1298(1)	22-1-14-109-12-4-31	NT (1)	*sea*(1)
	t3622	1(0.7)	ST88(1)	22-1-14-23-12-4-31	IV(1)	*sea*(1)
Singleton	t078	1(0.7)	NT(1)	1-1-4-1-new-new-1	III (1)	*seg*(1)
Unkown	t5554	1(0.7)	ST630 SLV	12-334-1-1-4-4-3	V(1)	None
	NT	1(0.7)	NT(1)	1-4-new-169-new-57-11	NT (1)	None

a CC: clone complex.

b Allelic profile in order of *arcC*-*aroE*-*glpF*-*gmk*-*pta*-*tpi*-*yqiL*.

SLV: single locus variant.

NT: not typeable, the reasons for the nontypeable of some isolates were mainly due to that a low quality of sequence chromatogram was present for *spa* typing, or at least one new allele was identified in the seven housekeeping genes for MLST typing, or no corresponding band was found in the multiplex PCR for SCC*mec* typing. The typing experiments were repeated at least twice for the nontypeable isolates.
