# Supplementary figures and images for: Characterization of Staphylococcus aureus from Distinct Geographic Locations in China: An Increasing Prevalence of spa-t030 and SCCmec Type III
Source: PLoS One. 2014 Apr 24;9(4):e96255. doi: 10.1371/journal.pone.0096255 (PMC3999196; doi:10.1371/journal.pone.0096255)

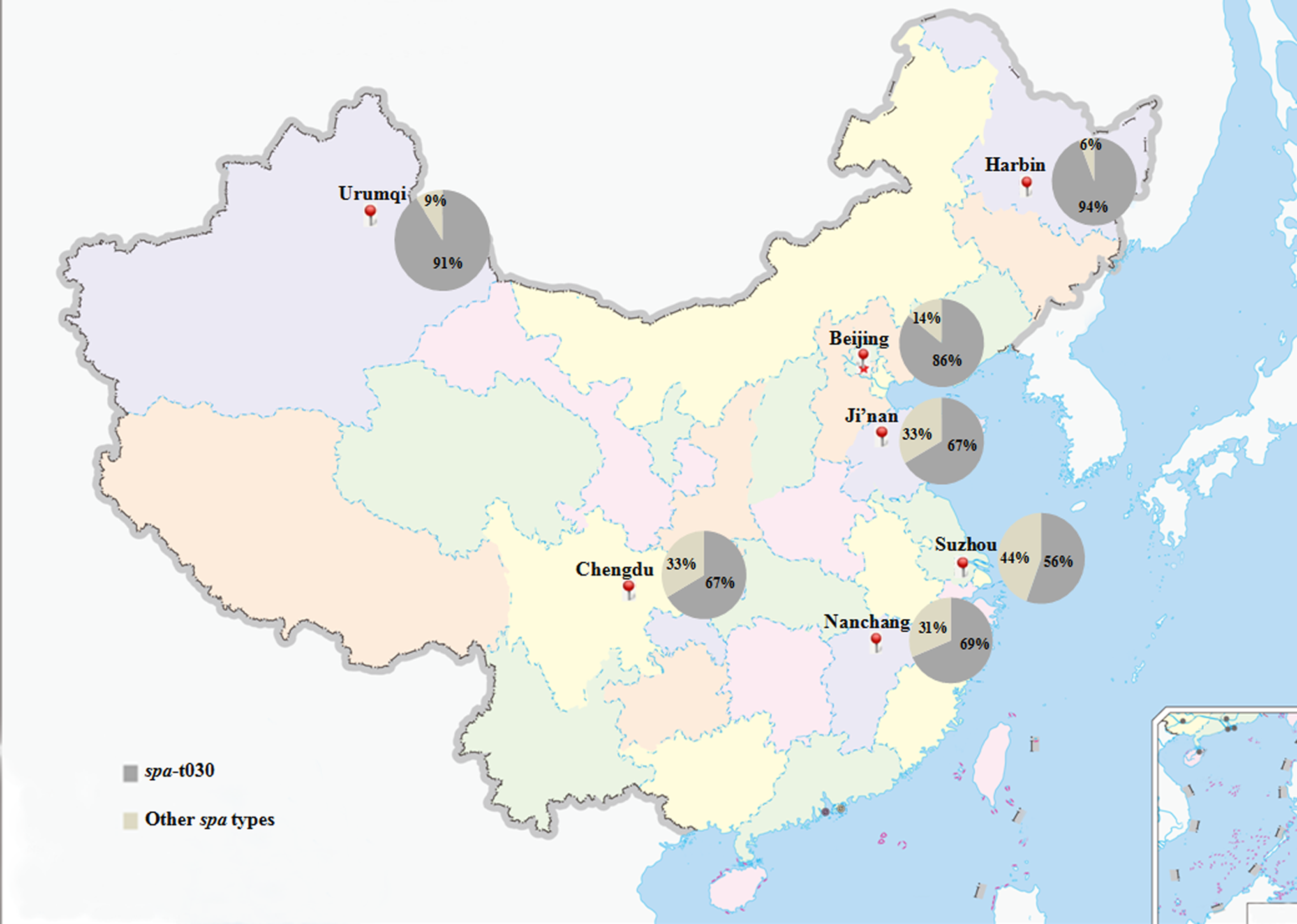

Supplement: Figure S1 — Locations of seven participating hospitals in China and the proportions of spa -t030 and other spa types among MRSA isolates in this study. (TIF) [file pone.0096255.s001.tif]
