# Supplementary material for: Characterization of Staphylococcus aureus from Distinct Geographic Locations in China: An Increasing Prevalence of spa-t030 and SCCmec Type III
Source: PLoS One. 2014 Apr 24;9(4):e96255. doi: 10.1371/journal.pone.0096255 (PMC3999196; doi:10.1371/journal.pone.0096255)
Supplement: Table S1 — The corresponding spa types, number of MRSA, virulence genes distribution and city distribution of 37 pvl-positive isolates according to different ST types. (DOCX) [file pone.0096255.s002.docx]

**Table S1. The corresponding *spa* types, number of MRSA, virulence genes distribution and city distribution of 37 *pvl*-positive isolates according to different ST types.**

| MLST | No.(%) of isolates | *spa* type (no. of isolates) | No. of MRSA | Virulence genes distribution (no. of isolates) | | | | | | | | | Distribution by city (no. of isolates) |
| --- | --- | --- | --- | --- | --- | --- | --- | --- | --- | --- | --- | --- | --- |
|  |  |  |  | *sea* | *seb* | *sec* | *sed* | *seg* | *she* | *sei* | *sej* | *tst* |  |
| ST22 | 10(27.0) | t309(9),t12442(1) | 0 | 8 | 0 | 0 | 1 | 8 | 0 | 3 | 0 | 0 | Ur(10) |
| ST398 | 8(21.6) | t011(3),t034(2),t189(1),t1451(1),t12441(1) | 0 | 4 | 0 | 0 | 0 | 1 | 0 | 0 | 0 | 0 | BJ(3),CD(1),HB(1),JN(1),NC(1), Ur(1) |
| ST59 | 6(16.2) | t437(5),t163(1) | 4 | 1 | 3 | 1 | 0 | 0 | 0 | 0 | 3 | 0 | JN(5),NC(1) |
| ST121 | 3(8.1) | t284(1),t308(1),t1994(1) | 0 | 3 | 1 | 0 | 0 | 0 | 0 | 0 | 0 | 0 | Ur(3) |
| ST188 | 2(5.4) | t189(2) | 0 | 2 | 2 | 0 | 0 | 0 | 0 | 0 | 0 | 0 | CD(2) |
| ST30 | 2(5.4) | t318(2) | 0 | 2 | 0 | 1 | 0 | 0 | 0 | 0 | 0 | 0 | JN(1),Ur(1) |
| ST7 | 1(2.7) | t796(1) | 0 | 0 | 0 | 0 | 0 | 0 | 0 |  | 0 | 0 | CD(1) |
| ST25 | 1(2.7) | t287(1) | 1 | 0 | 0 | 0 | 0 | 0 | 0 | 1 | 0 | 0 | CD(1) |
| ST943 | 1(2.7) | t289(1) | 0 | 0 | 0 | 0 | 0 | 0 | 0 | 1 | 0 | 0 | Ur(1) |
| ST2124 | 1(2.7) | t067(1) | 0 | 1 | 0 | 0 | 0 | 0 | 0 | 0 | 0 | 0 | Ur(1) |
| New^a^ | 1(2.7) | t758(1) | 0 | 0 | 0 | 0 | 0 | 1 | 0 | 0 | 1 | 0 | JN(1) |
| NT | 1(2.7) | t223(1) | 0 | 0 | 0 | 0 | 0 | 1 | 0 | 1 | 0 | 1 | Ur(1) |
| Total | 37(100.0) | - | 5 | 23 | 0 | 2 | 2 | 17 | 2 | 10 | 5 | 1 | - |

^a^ The allele profile of the new ST type was 6-5-16-2-7-14-15.

^b^ BJ, Beijing; CD, Chengdu; HB, Harbin; JN, Ji’nan; NC, Nanchang; SZ, Suzhou; Ur, Urumqi.

NT: not typeable.
