# Supplementary material for: Characterization of Staphylococcus aureus from Distinct Geographic Locations in China: An Increasing Prevalence of spa-t030 and SCCmec Type III
Source: PLoS One. 2014 Apr 24;9(4):e96255. doi: 10.1371/journal.pone.0096255 (PMC3999196; doi:10.1371/journal.pone.0096255)
Supplement: Table S2 — Comparisons of the proportion of antibiotic resistance and presence of virulence factors among epidemic MRSA clones, non-epidemic MRSA clones and MSSA isolates*. (DOCX) [file pone.0096255.s003.docx]

**Table S2. Comparisons of the proportion of antibiotic resistance and presence of virulence factors among epidemic MRSA clones, non-epidemic MRSA clones and MSSA isolates^*^.**

| Variables | | MRSA | | |  | MSSA (n=171) | *p* value^a^ | *p* value^b^ | *p* value^c^ |
| --- | --- | --- | --- | --- | --- | --- | --- | --- | --- |
|  |  | Epidemic clones  (n=130) | Non-epidemic clones  (n=21) | Total  (n=151) |  |  |  |  |  |
| Antibiotics, no. of resistance (%) | | | | | | | | | |
|  | P | 128(98.5) | 21(100.0) | 149(98.7) |  | 158(92.9) | 0.567 | 0.025 | 0.209 |
|  | E | 107(82.3) | 17(81.0) | 124(82.1) |  | 117(68.4) | 0.880 | 0.006 | 0.238 |
|  | CLI | 95(73.1) | 14(66.7) | 109(72.2) |  | 86(50.6) | 0.543 | <0.001 | 0.164 |
|  | RIF | 105(80.8) | 4(19.0) | 109(72.2) |  | 25(14.6) | <0.001 | <0.001 | 0.593 |
|  | CIP | 115(88.5) | 4(19.0) | 119(78.8) |  | 41(24.0) | <0.001 | <0.001 | 0.615 |
|  | GM | 109(84.5) | 7(33.3) | 116(77.3) |  | 70(41.2) | <0.001 | <0.001 | 0.489 |
|  | SXT | 76(58.5) | 5(23.8) | 81(53.6) |  | 68(39.8) | 0.003 | 0.001 | 0.155 |
|  | TET | 89(68.5) | 10(47.6) | 99(65.6) |  | 42(24.6) | 0.062 | <0.001 | 0.025 |
|  | FD | 9(7.6) | 3(15.0) | 12(8.6) |  | 9(5.4) | 0.380 | 0.464 | 0.124 |
| Virulence factors, no. of presence (%) | | | | | | | | | |
|  | *pvl* | 0(0.0) | 5(23.8) | 5(3.3) |  | 32(18.7) | <0.001 | <0.001 | 0.576 |
|  | *sea* | 118(90.8) | 6(28.6) | 124(82.1) |  | 100(58.5) | <0.001 | <0.001 | 0.009 |
|  | *seb* | 1(0.8) | 8(38.1) | 9(6.0) |  | 17(9.9) | <0.001 | 0.001 | 0.002 |
|  | *sec* | 3(2.3) | 4(19.0) | 7(4.6) |  | 16(9.4) | 0.008 | 0.013 | 0.170 |
|  | *sed* | 1(0.8) | 1(4.8) | 2(1.3) |  | 11(6.4) | 0.260 | 0.013 | 1.000 |
|  | *seg* | 8(6.2) | 5(23.8) | 13(8.6) |  | 61(35.7) | 0.020 | <0.001 | 0.280 |
|  | *seh* | 2(1.5) | 1(4.8) | 3(2.0) |  | 13(7.6) | 0.364 | 0.017 | 1.000 |
|  | *sei* | 2(1.6) | 4(19.0) | 6(4.0) |  | 48(28.1) | 0.004 | <0.001 | 0.380 |
|  | *sej* | 5(3.8) | 1(4.8) | 6(4.0) |  | 23(13.5) | 1.000 | 0.004 | 0.482 |
|  | *eta* | 0(0.0) | 0(0.0) | 0(0.0) |  | 10(5.8) | - | 0.006 | 0.605 |
|  | *etb* | 0(0.0) | 0(0.0) | 0(0.0) |  | 4(2.4) | - | 0.518 | 1.000 |
|  | *tst* | 1(0.8) | 4(19.0) | 5(3.3) |  | 6(3.5) | 0.001 | 0.145 | 0.015 |
|  | *sasX* | 5(3.8) | 0(0.0) | 5(3.3) |  | 0(0.0) | 1.000 | 0.014 | - |

*p* value^a^ : epidemic MRSA clones (all the isolates corresponding to ST239) VS non-epidemic MRSA clones;

*p* value^b^ : epidemic MRSA clones(all the isolates corresponding to ST239) VS MSSA;

*p* value^c^ : non-epidemic MRSA clones VS MSSA.

**^*^** Data missing for some isolates.

P, penicillin; E, erythromycin; CLI, clindamycin; RIF, rifampin; CIP, ciprofloxacin; GM, gentamicin; TET, tetracycline; SXT, trimethoprim-sulfamethoxazole; FD, flusidic acid.
